# Short-chain fructo-oligosaccharides modulate gut microbiota composition and metabolism: dose–response assessment in an *ex vivo* gut model

**DOI:** 10.1080/29933935.2026.2674335

**Published:** 2026-05-20

**Authors:** Christelle Bressuire, Florence Thirion, Laura Chiaravano, Serigne Inssa Ngom, Romane Marion, Marine Gilles, Benoît Quinquis, Elliot Mathieu, Magali Berland, Hervé M. Blottière, Cindy Le Bourgot, Christel Béra-Maillet

**Affiliations:** a Université Paris Saclay, INRAE, AgroParisTech, Micalis Institute, Jouy-en-Josas, France; b Université Paris-Saclay, INRAE, MGP, Jouy-en-Josas, France; c Tereos, Scientific and Regulatory Affairs Department, Moussy-le-Vieux, France

**Keywords:** Prebiotics, short-chain fructo-oligosaccharides, gut microbiota, fermenters, metagenomics

## Abstract

Short-chain fructo-oligosaccharides (scFOS) are prebiotic fiber rapidly fermented in the colon and known to stimulate beneficial bacteria, such as *Bifidobacterium* spp. and *Lactobacillaceae*. While their overall effects on the gut microbiota are established, the dose–response relationship remained only partially characterized. This study aimed to determine the minimum effective dose of scFOS required to modulate gut microbiota composition and functions. An *ex vivo* chemostat model was used to simulate colonic fermentation with different doses of scFOS (1 to 10 g/d). Microbiota composition and metabolic activity were assessed by qPCR, short-chain fatty acid (SCFA) quantification, and shotgun metagenomics. An increase in scFOS dose led to higher SCFA levels, particularly acetate and butyrate, along with a modification in microbial composition, with a minimum significant effective dose of 2.5 g/d. Significant increase in *Bifidobacterium adolescentis*, *Anaerostipes hadrus*, and *Clostridium innocuum* was observed at the same dose. Functional analysis revealed an enrichment of *GH32* genes in the pangenomes of species positively impacted by scFOS. These findings demonstrate that low doses of scFOS can effectively modulate the gut microbiota and enhance SCFA production, supporting their use in dietary interventions aimed at improving intestinal health.

## Introduction

The human gut microbiota is a complex ecosystem harboring trillions of microorganisms that contribute to essential functions in human physiology, including metabolism, immune modulation, and the maintenance of intestinal barrier integrity.^1^ ^,^ ^2^ Under healthy conditions, this diverse microbial community remains in dynamic equilibrium with the host. However, multiple environmental factors, notably diet, can alter its composition and metabolic outputs, potentially shifting the ecosystem toward dysbiosis, a state associated with various disorders, such as inflammatory bowel diseases, metabolic syndrome, and obesity.^3^ ^,^ ^4^ Among dietary factors, dietary fibers play a central role in shaping gut microbial communities. Fibers that escape digestion in the upper gastrointestinal tract reach the colon, where they are fermented by specific microbial populations, producing gases and metabolites, such as short-chain fatty acids (SCFAs), that influence host physiology.^5^ ^-^ ^7^ Increased dietary fiber intake has been linked to reduced risks of obesity, type 2 diabetes, and cardiovascular disease, highlighting the importance of microbiota-mediated fermentation in metabolic health.^8^ ^,^ ^9^

Within this context, prebiotic fibers represent a subset of dietary fibers of particular interest because they are selectively utilized by host microorganisms in ways that can support gut health. The International Scientific Association for Probiotics and Prebiotics (ISAPP) defines prebiotics as substrates that are selectively utilized by host microorganisms, conferring health benefits.^10^ Among these, short-chain fructo-oligosaccharides (scFOS) have gained considerable attention due to their well-documented bifidogenic effect and their ability to modulate gut microbial communities in both humans and animal models associated with health benefits.^11^ ^-^ ^15^

Structurally, scFOS derived from sucrose possess unique molecular characteristics that distinguish them from other prebiotic fibers, such as inulin or longer-chain FOS or oligofructose. Produced via enzymatic action, scFOS consist of a terminal glucose residue linked to short chains of fructose units via β(2→1) glycosidic bonds, typically with a degree of polymerization (DP) between 3 and 5. Unlike inulin and oligofructose, which often exhibit variable chain lengths, heterogeneous compositions, and fluctuating purity depending on botanical origin and obtention methods, scFOS from beet sucrose offers a defined and consistent structure. This molecular precision—combined with high purity and controlled oligosaccharide distribution—contributes to faster fermentation kinetics, compared to longer-chain FOS or inulin, and more targeted microbial modulation in the colon.^7^ ^,^ ^16^

Several human intervention studies have demonstrated that scFOS are associated with an increased abundance of *Bifidobacterium spp.*, accompanied by improvements in bowel function and potential benefits for glucose homeostasis.^11^ ^,^ ^13^ ^,^ ^17^ Moreover, recent evidence suggests that scFOS fermentation not only enhances *Bifidobacterium* populations but may also support the growth of butyrate-producing bacteria through metabolic cross-feeding mechanisms.^18^ ^,^ ^19^ Butyrate is recognized for its key roles in maintaining colonic epithelial integrity, exerting anti-inflammatory effects, and modulating immune responses.^18^ ^,^ ^20^ ^,^ ^21^

Despite the well-established benefits of scFOS, important knowledge gaps remain regarding its dose–response relationship and the minimum effective dose required to induce specific microbial and metabolic changes. Although scFOS are well tolerated and recognized as food ingredients meeting the definition of dietary fibers, it remains essential to determine the threshold at which significant modulation of the gut microbiota occurs. This is crucial for designing effective dietary interventions and optimizing fiber-enriched food products.^11^ ^,^ ^22^

Conducting dose–response studies directly in humans can be challenging due to the complex relationships between the microbiota and the host, which can limit the interpretation of microbiota responses to treatments. Additional constraints include ethical considerations, high costs, interindividual variability, and practical limitations in sample collection and control of external influences. In this context, *ex vivo* gut fermentation systems offer a valuable alternative for investigating microbiota responses to dietary fiber under standardized and highly controlled conditions. These models, particularly dynamic systems, such as chemostats, enable precise testing of substrate doses during fermentation kinetics while preserving key microbial interactions.^23^ ^-^ ^25^ Importantly, combining these *ex vivo* systems with advanced analytical techniques provides deeper insights into the taxonomic and functional changes induced by specific substrates. Shotgun metagenomics and metabolomics provide comprehensive profiling of microbial taxa, metabolic pathways, and bioactive compounds such as SCFAs.^26^ ^-^ ^29^ In association with *ex vivo* models, these tools offer a unique opportunity to decipher the mechanisms by which specific fiber doses affect microbial ecology and function.

However, while they reproduce several *in vivo* features, such as donor-specific microbial signatures, partial maintenance of species richness, and production of major metabolites like acetate, butyrate, and propionate, they do not fully capture physiological aspects of the human gut, including mucus layers, epithelial absorption, immune interactions, and the complex physical and chemical gradients present *in vivo*. Moreover, there is still no regulatory consensus on model selection or configuration, which may complicate interpretation and comparison across studies. Despite these limitations, *ex vivo* fermentations remain robust and reproducible tools to evaluate microbial and metabolic responses under controlled conditions, providing mechanistic insights that are often difficult to obtain directly in humans, and thus represent valuable complementary and potential alternative approaches to *in vivo* studies.^30^ ^-^ ^33^

The present study aimed to determine the minimum effective dose of scFOS capable of significantly modulating gut microbiota composition and metabolic activity under controlled *ex vivo* culture conditions. Using a continuous fermentation model inoculated with human fecal microbiota, we systematically evaluated the effects of scFOS at different doses (1 to 10 g/d) on microbial diversity, specific bacterial taxa, and SCFA production. Shifts in microbial gene content were analyzed using shotgun metagenomics, providing insight into the functional mechanisms underlying the observed taxonomic and metabolic changes. This research contributes to defining precise recommendations for the use of scFOS and supports the development of fiber-based strategies to modulate the gut microbiota for human health benefits.

## Materials and methods

### Ethics approval and consent to participate

The research protocol has been approved by the French ethics committee CPP Ile de France VI and registered under ID-RCB number 2022-A00367-36. The collection of stool samples had been obtained in accordance with data protection and legislation on civil liberties as stated by the National Commission of Computing and Freedoms (CNIL; France), based on the MR003 (Reference 2018-154_03/05/2018). All volunteers, fully informed of the objective of the project, have given a written consent confirming their willingness to participate before entry into the study.

### Human participants recruitment

For this study, recruitment of four French donors were based on the following characteristics: male or female adult (age 18—65), person with a fiber intake comprised between 15 and 20 g/d corresponding to the mean value of the French population (based on results from the INCA3 study, Anses 2017,^34^ and no use in 3 months prior to sampling of antibiotics and/or transit modulators (dietary fiber supplement, probiotics, prebiotics, synbiotics…) which could affect parameters followed during the study, no colonoscopy within the last 3 months and no individuals under legal protection (curatorship, guardianship, etc.).

### Preparation of Residue-free microbiota (RFM)

Immediately after emission, fresh stool (FS) was placed at 4 °C in a hermetic box under an anaerobic atmosphere, transported and processed in an anaerobic chamber (90% N_2_, 5% H_2,_ and 5% CO_2_) within 24 h. RFM was prepared from the fecal matrix (approximately 12 g of crude feces per donor) using the method of Juste et al.^35^ modified by Burz et al.^36^ with a preformed Optiprep^TM^ continuous density gradient (Stemcell technologies, Grenoble, France).^36^ It enables the recovery of live microbial cells that are free of organic and endogenous residues, unlike crude fecal suspensions, which also contain dead microorganisms. For the preformed gradient, sterile centrifuge tubes were filled with a degassed OptiPrep™ solution diluted in Hepes 10 mM–NaCl 9 g/L (pH 7), and stored vertically at –80 °C for at least 24 h before use; tubes were then thawed at room temperature to allow formation of a reproducible continuous density gradient. Stool suspensions were gently layered underneath this gradient and centrifuged at low temperature to allow bacterial cells to migrate to an intermediate phase while larger debris sedimented. Following centrifugation, the bacterial fraction was carefully collected, washed with Hepes–NaCl buffer to remove residual gradient material, and finally resuspended in sterile PBS for inoculation into the *ex vivo* fermenters. All steps except ultracentrifugation were performed under anaerobic conditions to preserve microbial viability and composition. Fecal samples are highly complex, containing bacteria, viruses, fungi, and endogenous debris, including undigested food. To study specifically the intestinal bacterial community, the main focus of our experiments, we performed a purification step to separate bacteria from other microorganisms and fecal residues, removing any undigested food or fibers that could interfere with subsequent experiments in the chemostat.

### Short-chain fructo-oligosaccharides characteristics

Fructo-oligosaccharides (FOS) used in this study are short-chain FOS Actilight^®^ 950P (Beghin-Meiji, France). Actilight^®^ 950P is obtained from sugar beet sucrose through an enzymatic reaction, with a proprietary fructosyltransferase enzyme. It consists of a terminal glucose unit (G) linked by an α(1→2) glycosidic bond to a short chain of β(2→1)-linked fructose units with varying degrees of polymerization (between 3 and 5). The result contains approximately 95% of scFOS, comprising a mixture of 37% ± 6% 1-kestose (GF2), 47% ± 6% nystose (GF3), and 16% ± 6% 1F-β-fructofuranosyl-nystose (GF4), and 5% of residual sugars (glucose, fructose, and sucrose). Four doses of Actilight^®^ 950P, readily soluble in water, were tested in this study: 1 g, 2.5 g, 5 g, and 10 g/d.

### *Ex vivo* continuous fermentations

**Figure 1.**
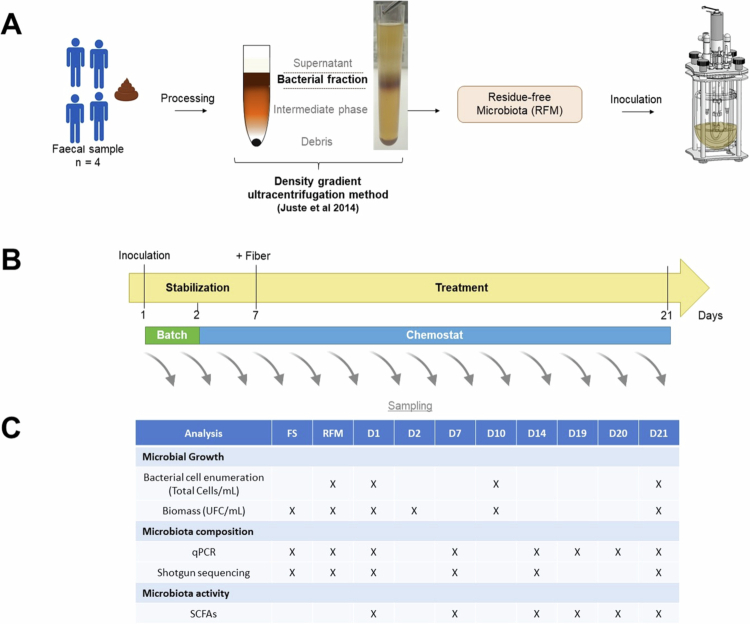
*Ex vivo* chemostat model for studying dose-dependent effects of scFOS on human gut microbiota. (A) Residue-free microbiota (RFM) was prepared from fresh human feces using a density-gradient ultracentrifugation method, as previously described,^35^ and used directly to inoculate five parallel fermentation vessels per donor. (B) Following a 24-h batch culture phase (to allow microbial adaptation and revivification), continuous fermentation was activated in chemostat mode with a constant flow of anaerobic basal medium. Environmental parameters (temperature, pH, redox potential, and stirring speed) were monitored and automatically controlled during the experiment. After a 7-d stabilization period, the treatment phase began, during which short-chain fructo-oligosaccharides (scFOS) were administered at daily doses of 1, 2.5, 5, or 10 g/d, split into two equal portions (morning and evening). For each donor, one vessel was kept as an untreated control (no fiber). (C) Microbial and metabolic analyses were performed on samples collected at different time points from day 1 to day 21.

Fermentations were conducted in Biobench fermenters (Biostream International, Doetinchem, The Netherlands), with five single vessels operating simultaneously, for each donor. Four vessels were reserved for the different doses of scFOS, and the fifth for the control condition (sterile water). This control fermenter was included to monitor baseline variation and spontaneous microbial shifts, ensuring that observed changes were due to scFOS supplementation rather than natural fluctuations of the microbiota. Experimental design is illustrated in Figure 1. Each donor was used for a single, independent experiment, providing four biologically independent replicates across the four donors per condition. The fermenters were fully computer-controlled and equipped with sensors for pH, redox potential, and temperature, which were continuously monitored (pH, temperature, stirring, redox...) to ensure stable and reproducible conditions. Vessels were filled with a basal fermentation medium adapted from Macfarlane et al.^37^ and composed as follows: peptone water (5 g/L), tryptone (5 g/L), yeast extract (4.5 g/L), starch (5 g/L), mucin (4 g/L), NaCl (4.5 g/L), KCl (4.5 g/L), casein (3 g/L), arabinogalacatan (2 g/L), pectin (2 g/L), NaHCO_3_ (1.5 g/L), MgSO_4_.7H_2_O (1.25 g/L), guar gum (1 g/L), Tween 80 (1 mL/L), L-cysteine (0.5 g/L), KH_2_PO_4_ (0.5 g/L), bile salt (0.4 g/L), CaCl_2_.6H_2_O (0.15 g/L), hemin (50 mg/L), FeSO_4_.7H_2_O (5 mg/L), and resazurin (1 mg/L). Vessels were then sterilized (121 °C, 15 min), cooled, and supplemented with a filter-sterilized vitamin solution (5 mL/L). This solution consisted of ascorbic acid (0.3 g/L), thiamine (0.01 g/L), riboflavin (0.01 g/L), and phylloquinone (0.003 g/L). The supplemented medium was gassed overnight with O_2_-free mixed gas Bio-300 (90% N_2_, 5% H_2_, 5% CO_2_; Air liquide, Grigny, France). Temperature was set at 37 °C and the stirring speed at 200 rpm. Then, the five vessels were simultaneously inoculated with RFM (approx. 9.5 × 10^6^ CFU/mL). Anaerobiosis was maintained with constant bubbling during the whole experiment (15 mL/h). After inoculation, a batch growth phase occurred for 24 h without pH control, to allow the microbiota to adapt to the system (Figure 1A). Thereafter, the fermenters were switched to chemostat mode with a constant working volume of 200 mL, supplied continuously with fresh supplemented anaerobic medium (maintained at 4 °C with stirring). We previously ensured that no bacterial growth occurred in any of these non-inoculated fiber-enriched media. Retention time in all systems was set at 24 h, and pH was maintained at 6.8 ± 0.4 by automatic adjustment using 3 M KOH. After a 7-d stabilization period monitored through SCFA and microbial analysis, the treatment phase was initiated (Figure 1B). Each scFOS daily dose (1, 2.5, 5, or 10 g/d) was divided into two equal portions, administered 6 h apart, and the treatment was applied for 14 d. During the whole process (21 d), each vessel was collected aseptically at several time points from day 1 (inoculation) to day 21 (Figure 1C) for analysis (8 mL). Throughout the experiment, enumeration of total bacteria was regularly monitored to check the stability of the microbial community, both during the initial 7-d stabilization period and the treatment phase, confirming that the chemostat had reached and maintained a steady state. Flow rates of fresh medium and waste removal were also controlled to ensure that the working volume and retention time were consistently maintained.

### Short-chain fatty acid

Briefly, the samples were centrifuged (10,000 × *g* for 5 min) and the bacterial supernatants were stored at −80 °C. Total SCFA concentrations were determined by gas chromatography performed by ICAN Omics Metabolomics (IHU, Paris, France). The presented values are the mean (±SEM) of independent measures in samples from the three technical replicates.

### Enumeration of total bacteria

Samples collected at days 1, 2, 10, and 21 were serially diluted in PBS solution before plating onto Brain Heart Infusion (BHI) agar medium (BD, Franklin Lakes, USA) supplemented with yeast extract 5 g/L, hemin 0.1% 10 mL/L, cellobiose 0.5 g/L, maltose 0.5 g/L, soluble starch 0.5 g/L, cysteine-HCl 0.5 g/L, and clarified rumen fluid 10%, in an anaerobic chamber (Bio-300). Duplicate plates from each dilution were placed in an anaerobic atmosphere using GENbag Anaer (Biomerieux, Craponne, France), transferred out of the anaerobic chamber and incubated at 37 °C for 48 h. Total bacterial enumerations were determined as the logarithmic value of the number of colony-forming units (CFU) per mL (log CFU/mL).

### Bacterial reference strains, DNA isolation, and quantitative PCR (qPCR)

Selected bacterial groups and species for this study are listed in Table 1. Bacteria were cultivated anaerobically on their appropriate medium and temperature, as recommended by DSMZ. For each strain, the total number of bacteria, in terms of CFU, was estimated by agar-plating. Three aliquots of 2 mL of overnight culture were centrifuged at 12,000 × *g* for 2 min and the bacterial pellets were stored at −20 °C before DNA isolation. Genomic DNA was isolated from each bacterial reference strain at the stationary phase of growth using the method described by Yu and Morrison et al.^38^ DNA extraction from FSs, RFMs, and all fermenter samples from the four donors was carried out according to the International Human Microbiome Standards (IHMS) SOP 07 V2 procedure (https://human-microbiome.org/index.php?id=Sop). DNA was quantitated using Qubit Fluorometric Quantitation (ThermoFisher Scientific, Waltham, USA) and qualified using DNA size profiling on a Fragment Analyzer (Agilent Technologies, Santa Clara, USA). The presence of inhibitors in each DNA sample, properly diluted, was estimated using Taqman^®^ Exogenous Internal Positive Control and the Taqman^®^ Universal PCR Master Mix kit according to the manufacturer's recommendation (Applied Biosystems, Foster City, USA). Primers used to quantify total bacteria and selected bacterial groups are described in Table 1, and were purchased from Eurofins Genomics (Eurofins, Ebersberg, Germany). Primer sequences issued from previous studies were checked for specificity by submitting the sequences to the Probe Match program provided by Ribosomal Database Project II.21 Primers^39^ and by comparison with sequences from 1900 species from the integrated catalog of human gut microbiome reference genes.^40^ Quantitative PCR assays were conducted on a StepOnePlus Real-Time PCR System (Applied Biosystems, Foster City, USA). Each reaction mixture (20 µL) was composed of 10 µL Fast SYBR^®^ Green Master 2× (Applied Biosystems, Foster City, USA), 0.5 µL of each specific primer at the final concentration of 0.25 µM and 5 µL of appropriate DNA dilutions. The amplification program consisted of one cycle at 95 °C for 20 s, 40 cycles of amplification (95 °C for 3 s, 60 °C for 30 s). The quantification of bacterial groups was done as described previously.^41^ Results are expressed as a mean of three replicates for each day.

**Table 1.** Bacterial groups or species studied and the corresponding primers used for quantitative PCR analysis.

Bacterial group	Referent strain	Sequence forward (5′-3′)	Sequence reverse (5′-3′)	References
All bacteria	*Escherichia coli BW25113*	CGG TGA ATA CGT TCC CGG	TAC GGC TAC CTT GTT ACG ACT T	[42]
*Bifidobacterium* spp.	*Bifidobacterium longum DSM 20219*	TCG CGT CYG GTG TGA AAG	CCA CAT CCA GCR TCC AC	[43]
*Blautia* spp.	*B. hydrogenotrophica DSM 10507*	CGC GTG AAG GAA GAA GTA TC	GAG CCT CAA CGT CAG TTA CC	[44]
*Lactobacillus*/*Leuconostoc*/Pediococcus*	*Lactobacillus reuterii DSM 32035*	AGC AGT AGG GAA TCT TCC A	CGC CAC TGG TGT TCY TCC ATA TA	[41]
*Bacteroides*/*Prevotella*	*B. thetaiotaomicron DSM 225*	GAA GGT CCC CCA CAT TG	CGC KAC TTG GCT GGT TCAG	[45]
*Clostridium Cluster IV*	*Clostridium Leptum DSM 753*	GGC GGC YTR CTG GGC TTT	ACC TTC CTC CGT TTT GTC AAC	[46]
*Akkermansia muciniphila*	*Akkermansia muciniphila DSM22959*	CAG CAC GTG AAG GTG GGG AC	ATT TGC TCG GCT TCA CAG CT	[47]
*Eubacterium rectale*	*Eubacterium rectale ATCC33656*	CAT TGC TTC TCG GTG CCG TC	ATT TGC TCG GCT TCA CAG CT	[48]
*Clostridium coccoides*	*Clostridium coccoïdes DSM 935*	AAA TGA CGG TAC CTG ACT AA	CTT TGA GTT TCA TTC TTG CGA A	[44]
*Roseburia inulinivorans*	*Roseburia inulinivorans DSM 16841*	CGK ACT AGA GTG TCG GAG G	GTC ATC TAG AGT GTC GGA GG	[46]

^*^ Simplified as *Lactobacillaceae.*

### Shotgun sequencing

DNA was extracted from FSs, RFMs, and fermenters (*n* = 72) following the MGP SOP v1 (https://mgps.eu/sops/mgp-sop-001-v1). For samples from fermenters and RFMs, the lysis step was performed directly on a pellet obtained from 2 mL of sample after centrifugation at 10,000 × *g* for 5 min. DNA size distribution was checked prior to sequencing by Fragment Analyzer (Agilent Technologies, Santa Clara, US) using the Genomic 50 kb kit. About 500 ng of DNA was fragmented by sonication using the E220 Focused-ultrasonicator (Covaris, Woburn, USA) and then underwent double purification using the DNA Clean Beads Kit (MGI Technology, Shenzhen, China) to select fragment sizes around 400 bp. About 100 ng of sized DNA was used to construct libraries using the Universal Library Prep kit and Universal Barcode Set (MGI Technology, Shenzhen, China). The amplified libraries (eight PCR cycles) were checked with the HS Small Fragment kit on Fragment Analyzer (Agilent Technologies, Santa Clara, USA) before being circularized and pooled. Circularized and pooled libraries at 50 pM were used to make DNA nanoball (DNB), which were loaded onto Flow Cell Large (FCL) flow cells and sequenced with the High Throughput sequencing kit PE150 (MGI Technology, Shenzhen, China) on a DNBseq G400RS platform (MGI Technology, Shenzhen, China) to obtain a minimum of 17.5 million of paired-end reads per sample (mean ± sd: 25 ± 5.3 million), except for three D1 samples (min–max: 0.6–13 million).

### Metagenomic profiling

Using fastp,^49^ low-quality reads were trimmed or filtered out. Remaining high-quality reads that successfully mapped on the human reference genome (T2T-CHM13v2.0) using bowtie2^50^ were further discarded. To account for differences in sequencing depth, 17.5 million read pairs (i.e. 35 million of single reads) were randomly selected for each sample with seqtk (https://github.com/lh3/seqtk). Then, abundance tables for species and carbohydrate-active enzymes (CAZYmes) encoding genes were generated with Meteor2,^51^ version 2.0.14, normalization = fpkm, references = human_gut and human_oral). References consist of microbial gene catalogs (10.4 million for the human_gut^52^ and 8.4 million for the human_oral),^53^ clustered into 1990 and 853 Metagenomic Species (MSP) with MSPminer,^40^ respectively, and annotated at the taxonomic level with the Genome Taxonomy Database (GTDB r220). Gene catalogs are also functionally annotated with dbcan3^54^ for CAZymes putative genes, providing direct insight into pangenome functional ability. Based on this annotation, Meteor2 computes the abundance of a given CAZyme in a sample as the sum of MSPs containing at least one gene annotated with that CAZyme gene, after restricting the pangenomes to the genes detected in the sample. Gut microbiota richness (or MSP richness) was computed as the number of MSP detected in a given sample (i.e. MSP whose abundance is not null). Cross-contamination between samples was checked on MSP abundance profiles with CroCoDeEL v1.0.3.^55^

### GH32 phylogenetic analysis

To predict the enzymatic activity of genes annotated as GH32 (β-2,1 versus β-2,6 linkage specificity), catalytic domains of the corresponding proteins were aligned with MAFFT^56^ v7.526, together with 14 characterized GH32 sequences retrieved from the Carbohydrate Active Enzymes database (https://www.cazy.org/GH32_characterized.html, accessed January 20, 2026)^57^ and having curated Swiss-Prot entries in UniProtKB. The alignment was further filtered using Goalign^58^ to remove positions with less than 50% informative sites. A maximum-likelihood phylogenetic tree was inferred from the filtered alignment using IQ-TREE v2.3.6 (parameters: -m MFP -bb 1000 -alrt 1000 -nt AUTO).^59^ The resulting tree was visualized using iTOL 7.4.2.^60^

### Statistical analysis

All analyses for quantitative metagenomics were performed with R (v4.4.1). To assess which metagenomics features were impacted by the different fermenter conditions, we used the R package nparLD v2.2 to perform nonparametric analysis of the longitudinal data. *p* values from the interaction of the factors “timepoint” (D7, D14, and D21) and “fermenter” (Control, 2.5 g/d, 5 g/d) were saved and corrected for multiple tests with the Benjamini–Hochberg procedure. Finally, log-fold change and associated Wilcoxon tests were computed for each bioreactor between D7 and D14 on the one hand, and D7 and D21 on the other hand. Log-fold change was computed as the median (over the four donors) log2-transformed ratio between the abundance of a metagenomic feature for a given donor at D14 or D21, and D7. Bray–Curtis dissimilarity and associated Permutational multivariate analysis of variance (PERMANOVA) were computed with the R package vegan (v2.6-6.1). Principal Coordinates Analysis (PCoA) on Bray–Curtis dissimilarity was performed with the R package ade4 (v1.7.22). All figures were drawn with the R packages ggplot2 (v3.5.1), ggpubr (v0.6.0), and cowplot (v1.1.3). For qPCR and metabolite analyses, statistical analysis was performed using GraphPad Prism® 10.4.0 software (GraphPad Software, La Jolla, CA, USA). Experimental data were analyzed either by one-way ANOVA followed, when appropriate, by Tukey's Honest Significant Difference (HSD) post-hoc test, or by Student's *t*-test for pairwise comparisons. Statistical significance was set at *p* < 0.05.

## Results

### Establishment and stabilization of a reproducible human gut microbiota model in *ex vivo* fermenters

A continuous *ex vivo* fermentation system was implemented to study the dose-dependent effects of scFOS on human gut microbiota (Figure 1, see Methods). The impact of four different doses of scFOS (0, 1, 2.5, 5, or 10 g/d) was studied on the gut microbiota from four donors. Variations in community composition and functions were monitored through microbial growth measurements, qPCR, shotgun sequencing, and metabolite analyses (Figure 1). Comparison of the initial microbiota (FS and RFM) of the four donors revealed that the FSs of donors 1 and 3 were dominated by *Bacillota_A* (formerly *Firmicutes*), while the FSs of donors 2 and 4 were dominated by *Bacteroidota* (formerly *Bacteroidetes*). Processing FSs into RFMs increased *Bacillota_A* and decreased *Bacteroidota*, leading RFM from all donors to be dominated by *Bacillota_A* (Supplementary Figure S1A). However, species richness (ranging from 214 to 438 depending on the donor) remained stable during the process (Supplementary Figure S1B). Moreover, PCoA based on Bray–Curtis dissimilarity of species profiles revealed a donor-driven clustering, indicating that the processing of FSs into RFMs preserved individual-specific microbial composition (Supplementary Figure S1C).

Following inoculation, bacterial growth was monitored to assess community establishment and stability prior to fiber supplementation. Total cell counting and biomass increased during the first days, reaching a steady state around 10 log CFU/mL at D2 for all donors (Supplementary Figure S2A).

Quantitative PCR analyses of key gut bacterial groups measured on the control fermenters revealed generally stable abundances after D7, with profiles specific to each donor (Supplementary Figure S2B).

As shown in Supplementary Figure S2C, the abundance of five major bacterial groups commonly found in the human gut (*Bacteroides/Prevotella, Clostridium cluster XIVa, Blautia spp., Bifidobacterium spp.,* and *Lactobacillaceae* ) served as a proxy for initial microbiota composition and confirmed stable microbial abundances starting at D7, across fermenters designated to receive different scFOS doses.

### Dose-dependent impact of scFOS on human gut microbiota composition and metabolite production

**Figure 2.**
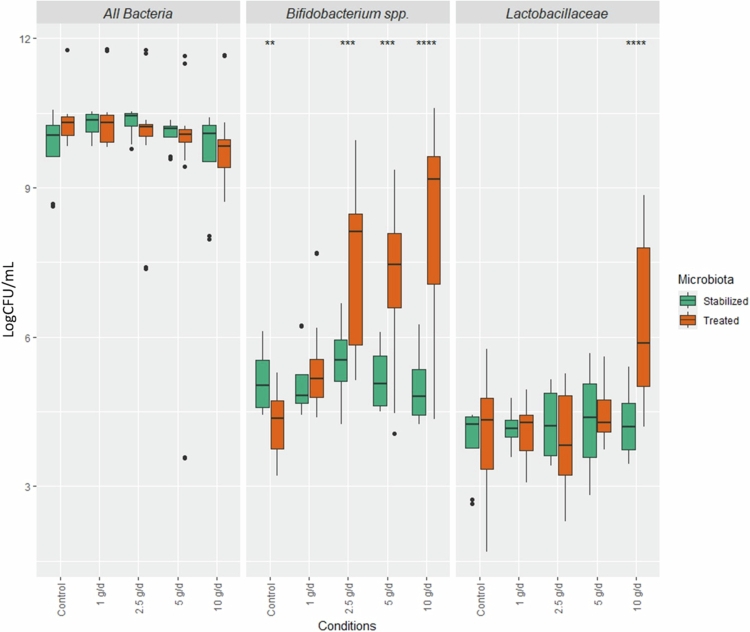
Quantitative response of gut-associated bacteria to increasing scFOS doses in *ex vivo* fermenters. Absolute abundance of *Bifidobacterium spp.,* *Lactobacillaceae* , and total bacteria quantified by qPCR. Data represent the mean values from the last 3 d of the treatment phase (days 19–21 = treated), compared to baseline (day 7 = stabilized). Statistical differences were evaluated using a *t* test. Bars show mean ± standard deviation. Significance levels are indicated as follows: *p* < 0.05 (*), *p* < 0.01 (**), and *p* < 0.001 (***).

Quantitative PCR analyses were conducted to assess the dose-dependent effects of scFOS supplementation on major gut bacterial groups (Figure 2). Data were pooled across all donors and compared between the stabilization phase (day 7) and the treatment phase (average of days 19–21).

For total bacterial counts, no significant differences were observed between stabilized and treated conditions across all tested scFOS doses.

In contrast, *Bifidobacterium spp.* showed a marked and dose-dependent response to scFOS. Specifically, *Bifidobacterium spp.* levels remained stable at the lowest dose (1 g/d) throughout the treatment phase, whereas higher doses (2.5, 5, and 10 g/d) resulted in significant increases in their abundance (3 to 5 log units). Notably, 2.5 g/d represented the lowest dose at which a clear bifidogenic effect was observed.

For *Lactobacillaceae*, abundance increased significantly at the highest dose of 10 g/d only, corresponding to approximately a 1 log increase. Other bacterial groups were also assessed but did not exhibit notable dose-dependent variations under these experimental conditions (Supplementary Figure S3).

Donor-specific analyses shown in Supplementary Figure S4 confirmed these pooled results. For *Bifidobacterium spp.,* the bifidogenic effect of scFOS was consistent across most donors, except for donor 2, who showed no response to the treatment, potentially because of low initial *Bifidobacterium spp*. abundance. For *Lactobacillaceae* , increases were mainly observed in donors 3 and 4 at higher doses, whereas donors 1 and 2 exhibited minimal or no response.

**Figure 3.**
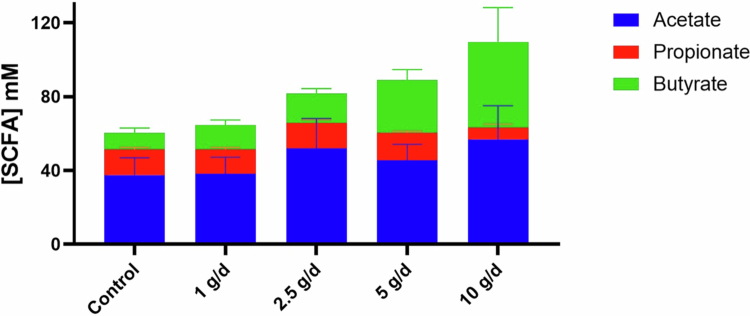
Dose-dependent increase in SCFA production following scFOS supplementation. Quantification of short-chain fatty acids (SCFAs): acetate, propionate, and butyrate; in the fermentation supernatants (days 19–21). Data are displayed as stacked bar plots to show the relative and total concentrations of individual SCFAs per condition expressed in mmol/L. SCFA concentrations were measured by gas chromatography, and results are presented as means ± SD of biological replicates and final days (*n* = 12).

SCFA concentrations were measured at the end of the treatment phase (days 19–21) to evaluate the functional consequences of scFOS fermentation (Figure 3). In pooled samples from all donors, the SCFA profile under control conditions (without fiber) exhibited an approximate ratio of 60% acetate, 20% butyrate, and 20% propionate. This ratio remained globally unchanged at the lowest dose of 1 g/d. However, starting at 2.5 g/d, clear shifts in SCFA production were observed. Both acetate and butyrate concentrations increased in a dose-dependent manner from 2.5 g/d, while propionate tended to decrease, particularly at the highest dose of 10 g/d. Donor-specific responses, presented in Supplementary Figure S5, confirmed the pooled trends, though notable inter-individual differences were observed. In particular, acetate increased in all donors from 2.5 g/d, whereas butyrate increased only in about half of them. In addition, the lowest dose of 1 g/d scFOS induced a slightly higher butyrate production in donor 4, not observed in other donors. Total SCFA concentrations at the end of the treatment phase were higher at increasing scFOS doses compared to the stabilization phase (Supplementary Figure S6). Overall, these results demonstrate a robust bifidogenic effect of scFOS from 2.5 g/d, associated with coordinated changes in microbial composition and metabolic activity.

### Impact of scFOS on gut microbiota composition

Since qPCR analysis revealed significant changes in *Bifidobacterium spp.* abundance and SCFAs production at doses as low as 2.5 g/d, we performed shotgun metagenomic sequencing on samples collected at D7, D14, and D21 from the control, 2.5 g/d and 5 g/d fermenters to gain insight into microbiota composition at species and strain level.

We first compared microbial communities at D7 with fresh stools and observed that, despite a reduction in richness and differences in species abundances as revealed by Bray-Curtis dissimilarity (Supplementary Figure S7A, B), D7 communities still reflected the original donor-specific microbiota. Indeed, D7 samples showed higher Spearman's correlation with their corresponding donor than with FS from other donors (Supplementary Figure S7C). Moreover, PCoA showed that D7 samples from the same donor clustered together (Supplementary Figure S7D), and consistently displayed lower Bray–Curtis dissimilarity and higher Spearman's correlation than D7 samples from different donors (Supplementary Figure S7E, F). Together, these results indicate that although substantial changes occur during the stabilization phase, microbial communities at D7 remain donor-dependent and retain a donor-specific signature.

**Figure 4.**
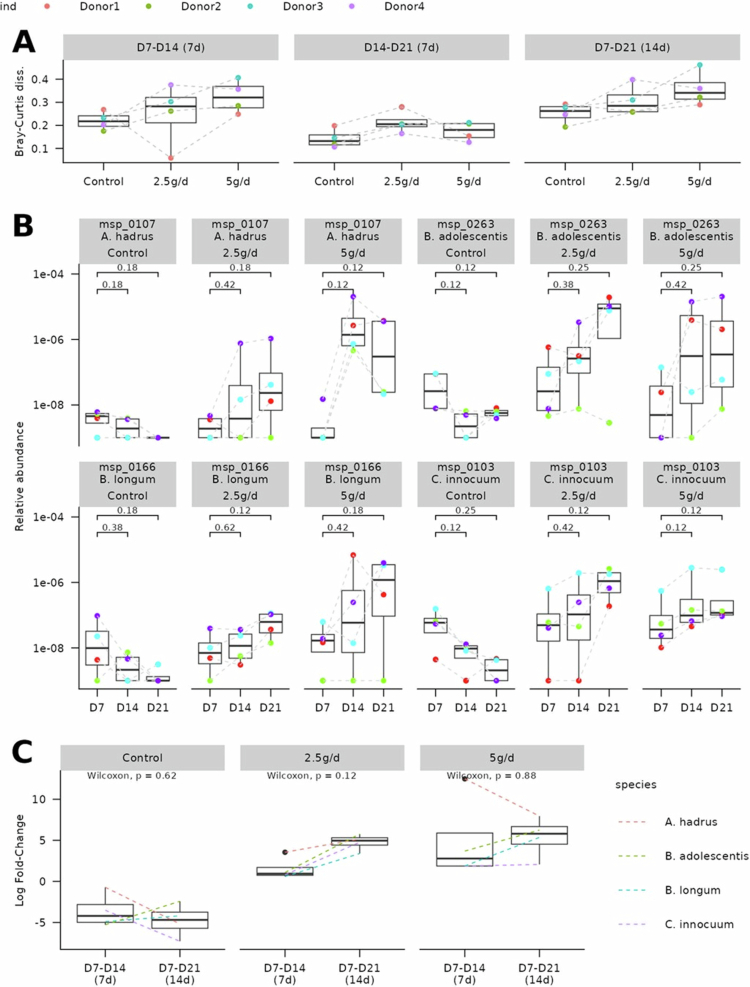
Impact of scFOS on gut microbiota composition: (A) Bray–Curtis dissimilarity calculated on log10-transformed species profiles between D7 and D14, D14 and D21, or D7 and D21, for each fermenter (control, 2.5 g/d or 5 g/d). Each point represents a donor, and dashed lines connect points from the same donor. (B) Relative abundance of the four species increased by scFOS at each time point in each fermenter. Dashed lines connect samples from the same donor. *p* values from Wilcoxon signed–rank tests between the control fermenter and other fermenters. (C) Log fold-change between D7 and D14 or D7 and D21 for the four species significantly increased by scFOS, in each fermenter. Dashed lines connect points corresponding to the same species. Log fold-change values represent the median over the four donors. *p* values from Wilcoxon signed–rank tests are displayed. *A. hadrus = Anaerostipes hadrus*, *B. adolescentis = Bifidobacterium adolescentis, B. longum = Bifidobacterium longum*, and *C. innocuum = Clostridium innocuum.*

Bray–Curtis dissimilarity analysis comparing D14 and D21 to D7 (baseline) confirmed that microbial composition was altered in both the 2.5 g/d and 5 g/d fermenters, related to control, already after 1 week of scFOS treatment (Figure 4A). Interestingly, Bray–Curtis dissimilarity between D7 and D14 was higher in the 5 g/d fermenter than in the 2.5 g/d fermenter, indicating a more rapid shift in microbial composition during the first week of treatment at the higher dose. However, this trend reversed during the second week (D14–D21), suggesting that although temporal dynamics differ, both doses may ultimately lead to a similar composition when reaching equilibrium.

At the species level, 17 species were significantly impacted by scFOS, as determined by a nonparametric longitudinal analysis testing the interaction between timepoint and fermenter conditions (*p* ≤ 0.05, Supplementary Table S1, see Methods). Among them, four species were increased in 2.5 g/d and 5 g/d fermenter (log fold change between D7 and D21 (D7–D21 logFC > 0) and decreased in control fermenter (D7–D21 logFC < 0): *Anaerostipes hadrus, Bifidobacterium adolescentis, Bifidobacterium longum,* and *Clostridium innocuum* (sorted by decreasing D7–D21 logFC in 5 g/d fermenter, Figure 4B). Consistent with the previous Bray–Curtis analysis, in the 2.5 g/d fermenter, the D7–D21 logFC of these species was higher than their D7–D14 logFC, whereas logFCs were similar in the 5 g/d fermenter, confirming that composition changes during the second week were more pronounced at the lower dose. In particular, in the 2.5 g/d fermenter, all species continued to grow, whereas in the 5 g/d fermenter, *C. innocuum* remained stable after D14, and *A. hadrus* even decreased (Figure 4C). Examination of individual donors revealed high inter-individual variability: for example, this pattern was evident in donor 1 but absent from donor 2 (Supplementary Figure S8). Confirming the qPCR results, *B. adolescentis* and *B. longum* showed little to no increase in donor 2, which may be explained by this donor having the lowest D7 abundance among the four donors (Figure 4B).

### Carbohydrate-active enzyme profiles of species impacted by scFOS

**Figure 5.**
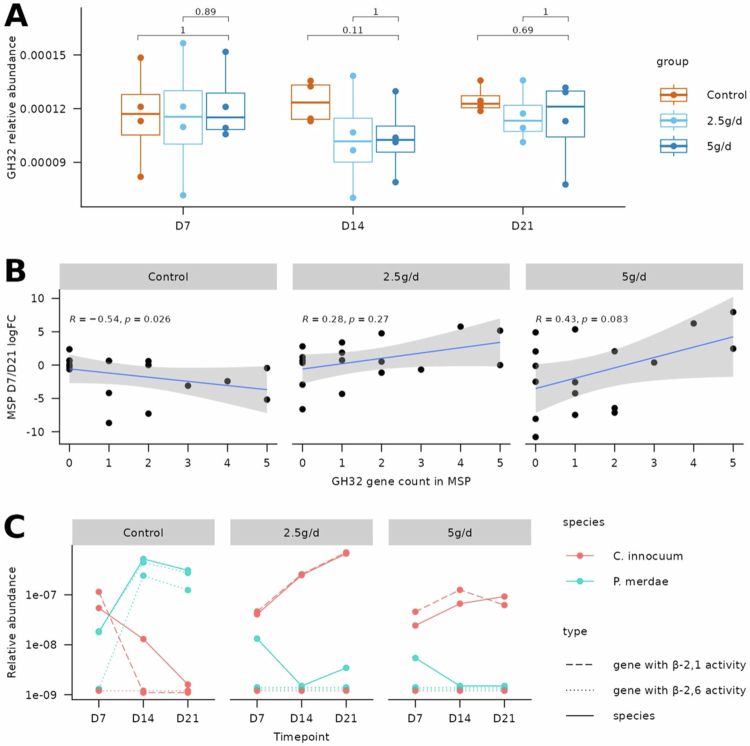
GH32 profiles at sample, species, and gene level: (A) GH32 potential in different fermenters and donors, at D7, D14, and D21. *p* values from Wilcoxon signed–rank tests are displayed. (B) Log fold-change between D7 and D21 of the 17 significantly impacted species, according to the number of genes annotated as GH32 in their pangenome. Log fold-change values represent the median over the four donors. Spearman's correlation coefficients along with their associated *p* value are displayed. (C) Relative abundance of two significantly impacted MSPs (*C. innocuum*: increasing and *P. merdae*: decreasing) and their respective GH32-annotated genes in the donor 4. Zero values (1e−09) were slightly offset to improve readability when multiple points with zero abundance overlapped.

The carbohydrate-active enzyme (CAZyme) GH32 is known to degrade FOS.^19^ ^,^ ^61^ Thus, we hypothesized that scFOS treatment may select species carrying GH32. However, we found no significant increase in GH32 potential (i.e. sum of species carrying GH32, see Methods) in 2.5 g/d or 5 g/d fermenters as compared to control (Figure 5A). We further investigated GH32 content of the 17 species impacted by scFOS by computing how many genes were annotated as GH32 in each species' pangenome. We observed that the association between GH32 gene count and the D7–D21 logFC increased with the scFOS dose: it was negative in the control fermenter (rho = −0.54), and became positive in the 2.5 g/d and the 5 g/d fermenters (rho = 0.28 and rho = 0.43, respectively, Figure 5B). The same pattern was consistently observed across individual donors (Supplementary Figure S9). Thus, an increased number of *GH32* genes was associated with increased logFC in 2.5 g/d and 5 g/d fermenters. These results suggest that a higher number of *GH32* genes in the species' pangenome may enhance their capacity to grow in response to scFOS treatment. For example, *A. hadrus*, the most significantly increased species, carries five genes annotated as GH32 in its pangenome.

However, some species carrying the same number of *GH32* genes did not respond similarly to scFOS treatment. As the GH32 family includes enzymes with distinct substrate specificities that are not necessarily active on the β-2,1 linkage characteristic of scFOS, we performed a phylogenetic analysis to predict *GH32* genes with β-2,1 versus β-2,6 linkage specificity (see Methods). Among the 29 GH32-annotated genes identified in the 17 MSPs of interest, 15 clustered with enzymes known to display β-2,6 linkage activity, whereas the remaining 14 genes grouped with characterized enzymes exhibiting β-2,1 linkage activity (Supplementary Figure S10). Importantly, the association between GH32 counts in MSP pangenomes and the D7/D21 logFC was strengthened when restricting the analysis to *GH32* genes with predicted β-2,1 linkage activity (rho = 0.38 and rho = 0.58 for the 2.5 g/d and 5 g/d fermenters, respectively, Supplementary Figure S11). Of note, these associations remained consistent across different clustering-based β-2,1 activity prediction criterion (data not shown).

Consistently, *Clostridium innocuum* and *Parabacteroides merdae* both harbor two *GH32* genes, yet exhibited opposite responses to scFOS supplementation. *C. innocuum*, which carries one *GH32* gene with predicted β-2,1 linkage activity, increased in abundance, whereas *P. merdae*, which lacks any *GH32* gene predicted to act on β-2,1 linkage, decreased. This pattern was observed in donor 4, irrespective of the total number of *GH32* genes present in the donor-specific strains (two in *P. merdae* and one in *C. innocuum*; Figure 5C). Together, these results indicate that β-2,1 linkage specificity, rather than total *GH32* gene count, is required for efficient scFOS utilization.

## Discussion

Short-chain fructo-oligosaccharides (scFOS) are well-recognized for their prebiotic properties, notably their ability to stimulate beneficial gut bacteria, such as *Bifidobacterium spp.,* and to influence metabolic output like SCFA production.^18^ ^,^ ^22^ ^,^ ^62^ In our *ex vivo* model, we demonstrated that scFOS supplementation induced clear dose–dependent effect on the composition and metabolic activity of human gut microbiota derived from healthy donors.

A fundamental condition of such studies is the establishment of a stable and representative microbial community. Given the high complexity of fecal samples, which include bacteria, viruses, fungi, and endogenous debris, such as undigested food, a purification step to generate Residue-Free Microbiota (RFM) was implemented to focus specifically on the intestinal bacterial community. This approach reduced the contribution of nonbacterial components and residual fecal material in the *ex vivo* system. Although the RFM differ from the original fecal samples, the four donors still maintain distinct community profiles, indicating that the separation process only minimally alters overall microbiota composition. Following inoculation into fermenters, the microbiota undergoes adaptation to the *ex vivo* environment, during which some loss of species richness is commonly observed, while the dominant community structure remains largely stable, as widely reported in the literature.^32^ ^,^ ^33^ ^,^ ^63^ In our study, we confirmed that despite these changes, day‑7 communities retain donor-specific signatures, with intradonor similarity consistently higher than interdonor similarity (Supplementary Figure S7), indicating that the microbiota composition remains sufficiently representative of the original fecal profiles for subsequent analyses. For a given donor, fermenters harbored nearly identical microbial communities prior to fiber treatment initiation, ensuring consistent baseline conditions for future comparison. Taken together, these results confirm that our *ex vivo* fermentation system establishes a stable and reproducible gut microbiota community, offering a robust and controlled model to investigate the effect of fiber treatment. The modulation of the gut microbiota by functional food is frequently addressed in the scientific community using *in vitro* or *ex vivo* studies,^30^ ^,^ ^32^ ^,^ ^64^ in order to identify functional food prototypes capable of modulating microbial composition and metabolite production, sometimes in a predictive manner. These studies confirm the physiological relevance of well-controlled *ex vivo* fermentations.

A consistent observation in our study is the bifidogenic effect of scFOS, which was already clearly observed at the low daily dose of 2.5 g. Importantly, this effect in *ex vivo* fermenters is consistent with clinical studies in healthy humans, which reported significant increases in fecal Bifidobacteria at doses starting from 2.5 g/d.^22^ ^,^ ^65^ ^,^ ^66^ Several human studies have tested daily scFOS intakes ranging from 2.5 to 10 g, showing dose-dependent increases in Bifidobacteria while maintaining good gastrointestinal tolerance. Clinical data further indicate that scFOS confer microbiota-related benefits even at low doses, including 5 g/d, and remain well tolerated at substantially higher intakes, up to 40 g/d.^67^ Another study has investigated intermediate daily intake of 20 g/d, providing complementary evidence of dose-dependent effects on the gut microbiota.^68^ From a translational perspective, the dose shown to be effective in our *ex vivo* model therefore corresponds to intake levels that are realistic for human nutrition and food applications, supporting both the safety and practical relevance of the doses identified in the present study.^22^ Interestingly, we note a decrease in *Bifidobacterium spp*. levels over time in control fermenters, suggesting that overall bacterial density remained stable throughout the experiment, but that specific bacteria may require fermentable substrates to maintain their abundance. This decline was prevented with scFOS supplementation, confirming the prebiotic potential of scFOS and its role in sustaining beneficial gut microbiota.

A significant metabolic impact of scFOS observed in our study was the dose-dependent increase in acetate and butyrate production, noticeable from 2.5 g/d, further supporting the dose-dependent fermentative activity of scFOS. The rise in butyrate level suggests possible metabolic cross-feeding interactions, wherein primary degraders of scFOS, such as *Bifidobacterium* spp., produce intermediates, such as acetate or lactate, subsequently utilized by secondary butyrogenic bacteria like *Faecalibacterium prausnitzii.* ^18^ ^,^ ^20^ This aligns with previous observations indicating that prebiotic fibers can indirectly stimulate butyrate production through such trophic interactions.^18^ Interestingly, propionate concentrations tended to decrease at higher scFOS doses, indicating a dose-dependent effect but requiring higher scFOS concentrations to achieve measurable changes, and possibly reflecting shifts in microbial metabolic pathways or competitive substrate utilization. Such changes in SCFA profiles could have physiological implications given the distinct roles of SCFAs in host metabolism and immune modulation.^18^ ^,^ ^69-72^ In particular, butyrate is a key energy source for colonocytes, promotes gut barrier integrity, and has anti-inflammatory properties, as shown in human and animal studies.^73^ ^-^ ^77^ Acetate, on the other hand, acts systemically by entering the circulation and modulating metabolic and hormonal pathways.^78^ ^-^ ^80^ It can influence insulin secretion, appetite regulation, and energy homeostasis. These findings underscore that even modest shifts in SCFA production, as observed in our *ex vivo* model, could have meaningful physiological effects if translated *in vivo*.

Since 2.5 g/d was the lowest dose showing a clear bifidogenic effect, and SCFA concentrations were minimal below this level, we focused our shotgun metagenomic analyses on 2.5 g/d and 5 g/d conditions. These doses were selected because they represented the threshold and next higher level where consistent microbial and metabolic shifts were observed, while still within ranges relevant for potential human supplementation.

Metagenomic analyses confirmed the increase in *Bifidobacterium* spp., by revealing that two different species, *B. adolescentis* and *B. longum,* were significantly enriched following scFOS supplementation. Beyond *Bifidobacterium spp*., our data indicate that scFOS can influence other bacterial species, including *Anaerostipes hadrus* and *Clostridium innocuum.* The first one is a known butyrate producer^19^ ^,^ ^81^ and was already found to degrade FOS with extracellular GH32 enzymes.^19^ ^,^ ^61^ ^,^ ^82^ It may contribute to the health-promoting effects associated with increased butyrate levels, such as anti-inflammatory properties and maintenance of colonic epithelial integrity.^18^ Notably, *A. hadrus* has also been reported to be more abundant in healthy individuals compared to those with metabolic disorders, which may suggest a positive role in gut microbiota modulation.^83^ In contrast, *Clostridium innocuum*, which also carries *GH32* genes, presents a more complex profile. While commonly detected in healthy gut microbiota,^69^ ^,^ ^84^ recent reports have linked it to opportunistic pathogenicity and potential disruptions in host metabolism.^85^ ^,^ ^86^ In our study, scFOS supplementation appeared to modulate the abundance of *C. innocuum*, but further work is required to clarify its role in fiber-driven microbiota changes.

Our data also revealed substantial interindividual variability in microbial responses to scFOS. For instance, donor 2 displayed a blunted bifidogenic response and minimal changes in SCFA production even at higher doses, whereas other donors exhibited pronounced shifts. Such donor-specific responses suggest that the capacity of the gut microbiota to respond to scFOS is strongly conditioned by baseline ecosystem features rather than by scFOS dose alone. Such variability is consistent with the notion that baseline microbiota composition significantly influences the response to prebiotic intervention.^22^ ^,^ ^69^ In particular, differences in baseline abundance of Bifidobacteria or butyrate-producing bacteria may explain why some individuals show strong bifidogenic and SCFA responses, whereas others respond only modestly to scFOS supplementation. Beyond species-level composition, strain-specific gene content is key to understanding the mechanisms underlying and predicting microbiota responses to prebiotic interventions.^87^ In this study, we found that the number of *GH32* genes—encoding carbohydrate-active enzymes involved in FOS degradation—was associated with the magnitude of the treatment effect on species abundance. The association was even stronger when restricting the analysis to *GH32* genes predicted to have β-2,1 linkage activity. This suggests that functional capacity for scFOS utilization, rather than taxonomic identity alone, determines the intensity of bifidogenic responses and downstream SCFA production. This observation could be explained by the broad enzymatic activities of GH32 enzymes, as species harboring more GH32 copies have a higher chance of including enzymes with higher affinity for the scFOS. However, differences in response both between and within species may depend on other factors, including the species baseline abundance, extra- or intracellular activity of GH32, which impacts cross-feeding,^19^ or other genes such as transporters.

Moreover, habitual fiber intake has been shown to modulate responsiveness to prebiotics, with individuals consuming higher dietary fiber exhibiting attenuated microbiota shifts in response to supplementation.^88^ All donors were selected to have a comparable daily fiber intake as part of the inclusion criteria, yet some individuals still exhibited a reduced bifidogenic and SCFA response, suggesting that microbiota may differ in their functional capacity or adaptation to fermentable substrates. While personalized dietary strategies are increasingly discussed, further work is needed to identify predictive markers of response and to optimize prebiotic use across diverse populations without necessarily requiring full personalization in all context.

A key strength of our study is the use of a continuous *ex vivo* fermentation model that enables controlled, reproducible investigation of gut microbiota dynamics under different fiber exposures. Our model maintained donor-specific microbial signature, consistent with earlier studies demonstrating the capacity of *ex vivo* systems to replicate key features of human gut microbiota.^18^ However, some limitations must be acknowledged. The 15-d fiber supplementation period, while adequate for observing initial microbial and metabolic changes, may not fully capture long-term adaptation or shift in stability of microbiota. Indeed, it was reported previously in rats that prolonged intake of FOS resulted in a short-term modification of gut microbiota that was not seen anymore after 8 and 27 weeks.^89^ Furthermore, spontaneous fluctuations observed in control fermenters underscore the dynamic nature of gut microbiota even in controlled systems, highlighting the necessity of rigorous control and sufficient replicates. The relatively small number of donors limits broader generalization, and future studies with larger cohorts are warranted to validate our findings and better capture inter-individual variability by investigating gene content of responding species at the strain level. One important limitation of *ex vivo* models is the absence of host factors, including epithelial absorption, immune signaling, and systemic metabolism. In our study, this prevented direct evaluation of the physiological consequences of microbial and metabolic changes on the host, including potential effects on gut barrier function, immune interactions, or systemic metabolism. Similarly, interactions between the microbiota and the host immune system, which can influence microbial composition and activity *in vivo*, were not observed. Despite these limitations, the controlled *ex vivo* setting allowed us to precisely evaluate microbial and metabolic responses to different scFOS doses, isolating the effect of the substrate itself. It should be noted that our study focuses specifically on microbial and metabolic responses in a controlled *ex vivo* environment, rather than attempting to capture the full complexity of host–microbiota interactions observed in animal models or human trials.

Overall, our study demonstrates that scFOS induces dose-dependent modulation of human gut microbiota composition and metabolism in an *ex vivo* setting. These findings highlight the potential of scFOS to promote beneficial bacterial taxa and enhance butyrate production at low doses, such as 2.5 g/d, easily incorporated into food formulations, with a rapid onset of action observed within just 7 d, suggesting possible contributions to host health. However, inter-individual variability showed the need for personalized approaches and for clinical studies to confirm the *in vivo* relevance of these observations. Future research should focus on elucidating species-specific mechanisms underlying fiber metabolism and identifying predictive markers of response to optimize the use of prebiotics in diverse populations.

## Supplementary Material

Bressuire_et_al_TableS1 Bressuire_et_al_TableS1

Supplementary_figures Supplementary_figures

## Data Availability

The data for this study have been deposited in the European Nucleotide Archive (ENA) at EMBL-EBI under accession number PRJEB101198 (https://www.ebi.ac.uk/ena/browser/view/PRJEB101198).
