# Supplementary material for: Short-chain fructo-oligosaccharides modulate gut microbiota composition and metabolism: dose–response assessment in an ex vivo gut model
Source: Gut Microbes Rep. 2026 May 20;3(1):2674335. doi: 10.1080/29933935.2026.2674335 (PMC13196643; doi:10.1080/29933935.2026.2674335)
Supplement: Bressuire_et_al_TableS1 [file KGMR_A_2674335_SM7387.docx]

**Table S1: List of metagenomic species with significant changes in time and fermenter**

| **msp_name** | **species** | **p** | **q** | **LogFC Control D7/D21** | **LogFC 2.5g/d D7/D21** | **LogFC 5g/d D7/D21** |
| --- | --- | --- | --- | --- | --- | --- |
| msp_0103 | *Clostridium_AQ innocuum* | 5.5e-07 | 5.3e-05 | -4.45 | 4.72 | 2.23 |
| msp_0107 | *Anaerostipes hadrus* | 5.5e-05 | 2.7e-03 | -1.73 | 4.01 | 7.44 |
| msp_0027 | *Parabacteroides merdae* | 1.5e-03 | 4.8e-02 | 1.41 | -2.08 | -4.07 |
| msp_0110 | *Bilophila wadsworthia* | 4.7e-03 | 1.1e-01 | 0.64 | -2.65 | -2.53 |
| msp_0263 | *Bifidobacterium adolescentis* | 6.3e-03 | 1.2e-01 | -2.41 | 5.29 | 5.71 |
| msp_0020 | *Enterocloster clostridioformis* | 1.2e-02 | 1.9e-01 | -0.49 | -0.01 | 1.77 |
| msp_0166 | *Bifidobacterium longum* | 2.3e-02 | 2.5e-01 | -3.06 | 3.07 | 4.62 |
| msp_1244 | *Collinsella sp. bacterium* | 2.3e-02 | 2.5e-01 | -4.97 | 1.32 | -2.77 |
| msp_0050 | *Anaerobutyricum hallii* | 2.4e-02 | 2.5e-01 | 0.00 | 0.84 | -2.93 |
| msp_0977 | *Lawsonibacter aceti* | 2.8e-02 | 2.6e-01 | 0.31 | -4.32 | -4.60 |
| msp_0509 | *Evtepia gabavorous* | 2.9e-02 | 2.6e-01 | 3.27 | 1.93 | -6.46 |
| msp_1291 | *Lentihominibacter faecis* | 3.5e-02 | 2.7e-01 | -1.55 | 1.65 | -0.20 |
| msp_0315 | *Fimisoma sp000435715* | 4.2e-02 | 2.7e-01 | 2.10 | 2.58 | 2.18 |
| msp_0772 | *Enterocloster pacaense* | 4.3e-02 | 2.7e-01 | -1.84 | -0.75 | -0.98 |
| msp_0346 | *Otoolea fessa* | 4.3e-02 | 2.7e-01 | 2.51 | 1.79 | -1.85 |
| msp_1403 | *Blautia_A ammoniilytica* | 4.6e-02 | 2.7e-01 | 1.75 | -3.02 | -5.34 |
| msp_0036 | *Bacteroides eggerthii* | 4.8e-02 | 2.7e-01 | -0.22 | 0.14 | -0.61 |
