# Supplementary material for: Short-chain fructo-oligosaccharides modulate gut microbiota composition and metabolism: dose–response assessment in an ex vivo gut model
Source: Gut Microbes Rep. 2026 May 20;3(1):2674335. doi: 10.1080/29933935.2026.2674335 (PMC13196643; doi:10.1080/29933935.2026.2674335)
Supplement: Supplementary_figures [file KGMR_A_2674335_SM7377.docx]

**
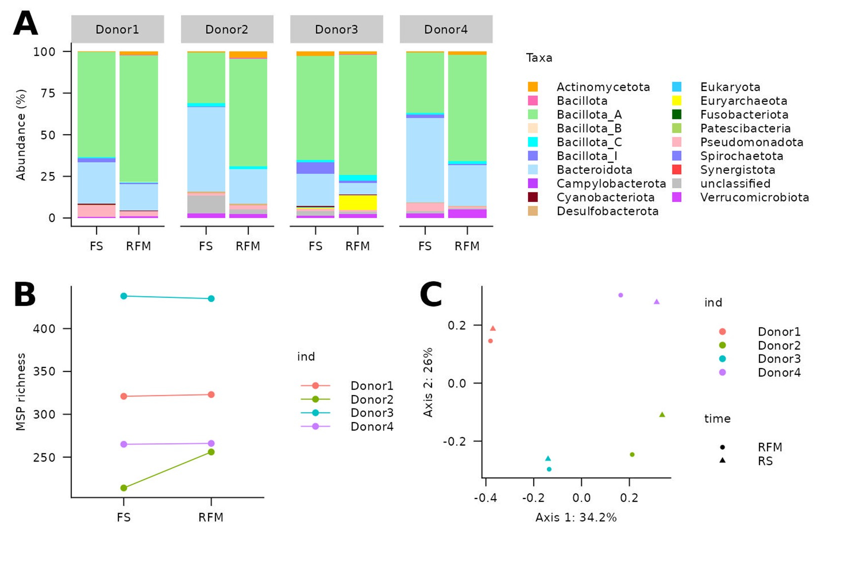
Figure S1:** Comparison of faecal samples and residue-free microbiota using shotgun metagenomic sequencing.

(A) Relative abundances of different microbial phyla. (B) Richness of FSs and RFMs, colored according to individuals. (C) Principal Coordinates Analysis (PCoA) based on Bray-Curtis dissimilarity matrix, computed on the Metagenomic Species (MSP) table for RFM and FS.

**
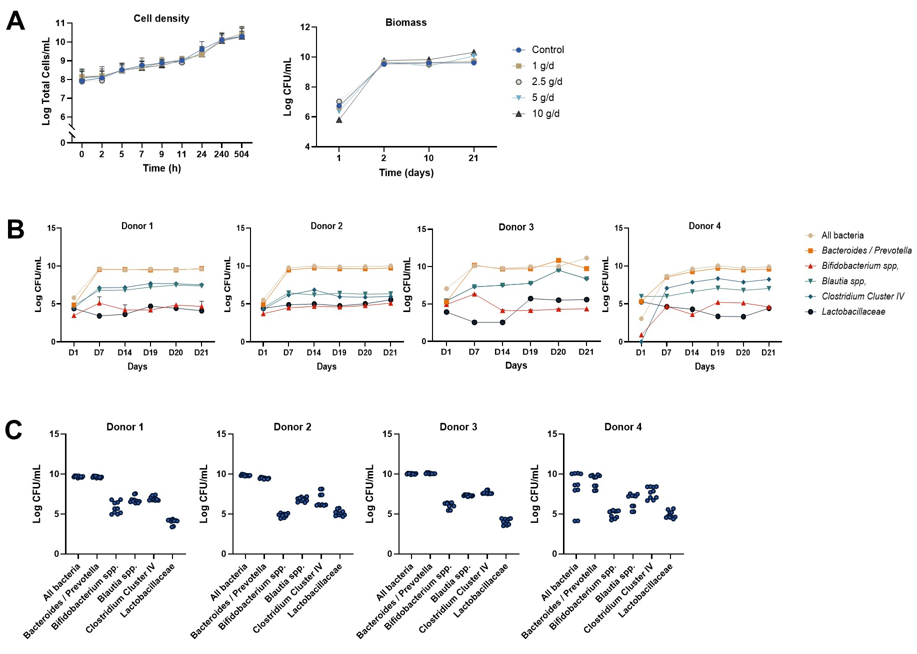
**

**Figure S2:** Bacterial quantification and validation of microbial stability in *ex vivo* fermenters prior to fibre treatment.

(A) Total cell density (Log Total cells/mL) and biomass (Log CFU/mL) measured under all experimental conditions. (B) Quantification by quantitative PCR (qPCR) of dominant bacterial groups commonly found in the human gut including: *Bacteroides/Prevotella*, *Clostridium cluster XIVa, Blautia spp., Bifidobacterium spp., and Lactobacillaceae spp.,* monitored over time in control fermenters for each donor. (C) Microbial abundances at day 7 (treatment baseline) in fermenters designated to receive different fibre doses. For each donor, qPCR was performed on each fermenter in technical replicates. Each point represents an individual qPCR measurement. The five bacterial groups monitored were *Bacteroides/Prevotella, Clostridium cluster XIVa, Blautia spp., Bifidobacterium spp.,* and Lactobacillaceae spp..

**
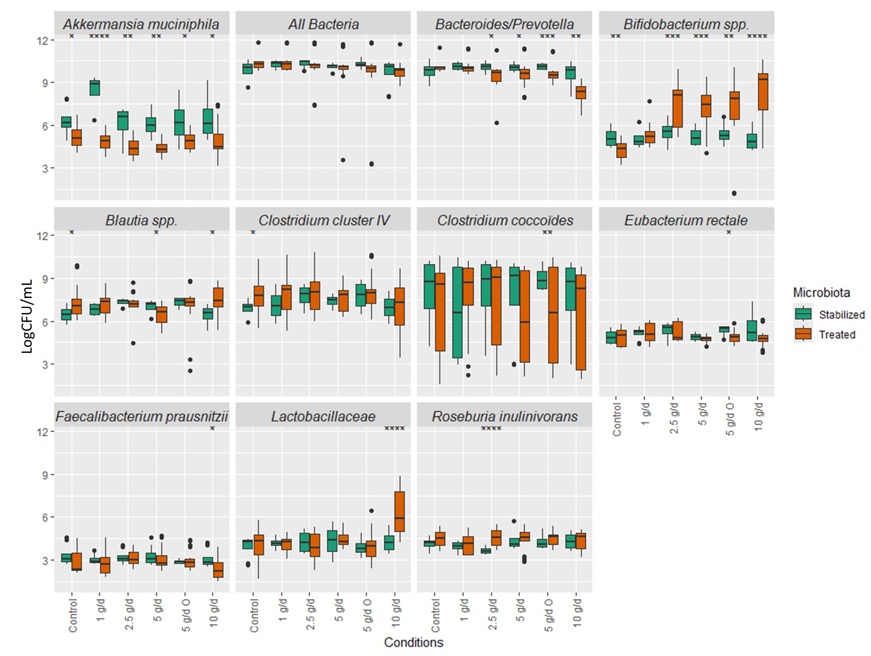
**

**Figure S3:** Quantitative response of other gut-associated bacteria tested in *ex vivo* fermenters

Absolute abundance of *Bacteroides/Prevotella, Clostridium cluster XIVa, Blautia spp.,* *Akkermancia muciniphila, Eubacterium rectale, Clostridium coccoides* and *Roseburia inulivorans* quantified by qPCR. Data represent the mean values from the last three days of the treatment phase (days 19-21= treated), compared to baseline (day 7=stabilized). Statistical differences were evaluated using a t-test. Bars show mean ± standard deviation. Significance levels are indicated as follows: *p* < 0.05 (*), *p* < 0.01 (**), *p* < 0.001 (***).

**
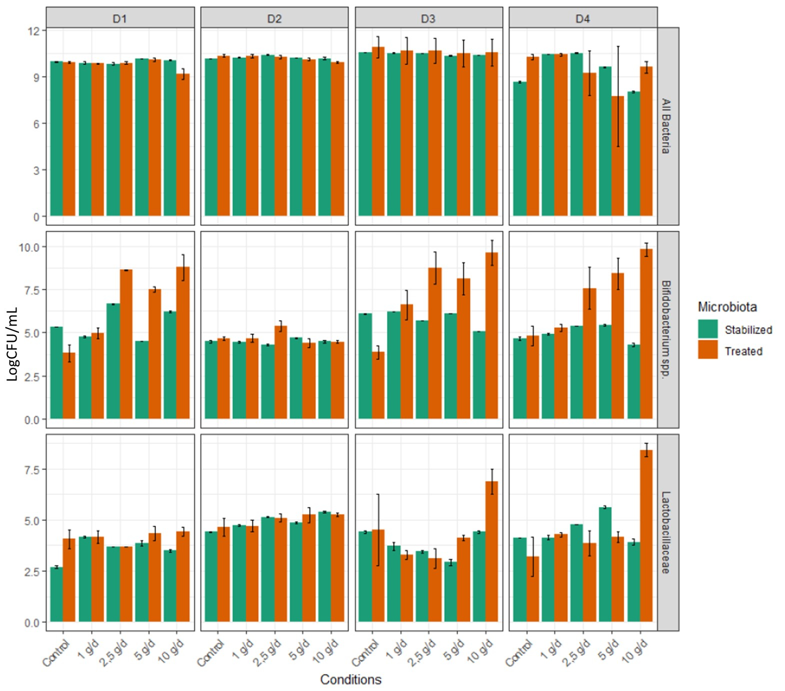
**

**Figure S4:** Donor-independent quantitative response of gut microbiota to increasing scFOS concentrations in *ex vivo* fermenters.

Absolute abundance of *Bifidobacterium spp*., Lactobacillaceae spp., and total bacteria quantified by qPCR, presented individually for each donor. Data represent values from the last three days of the treatment phase (days 19–21), compared to baseline (day 7). Statistical differences were evaluated using a paired t-test for each donor. Bars show mean ± standard deviation. Significance levels are indicated as follows: *p* < 0.05 (*), p < 0.01 (****), *p* < 0.001 (***).**

**
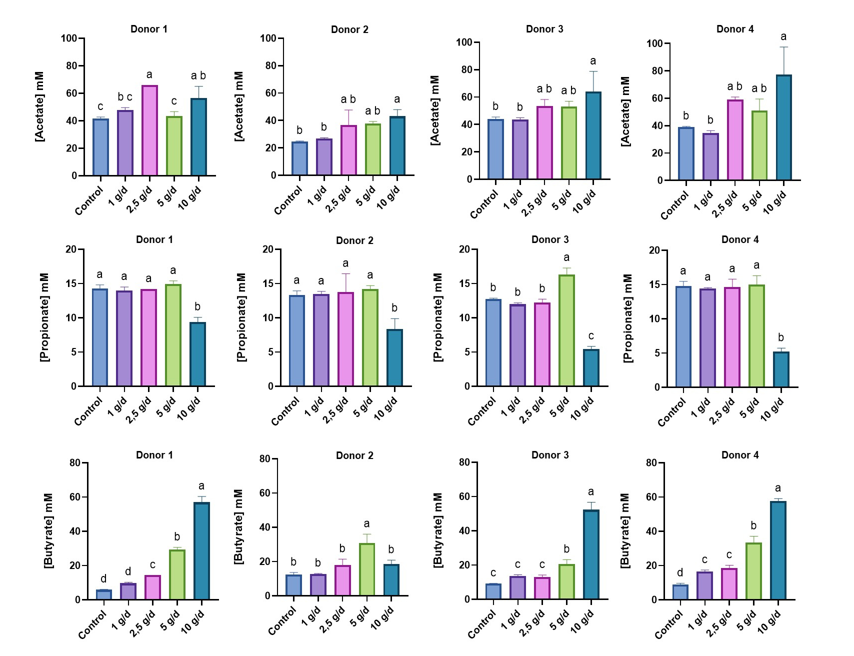
**

**Figure S5:** Donor-specific responses in individual SCFA concentrations following scFOS dose.

Concentrations of acetate, propionate, and butyrate (in mmol/L) measured at the end of treatment (days 19-21) shown separately for each donor and condition. Bars represent mean values. Statistical differences among conditions were assessed using one-way ANOVA followed by Tukey's HSD post-hoc test; conditions not sharing the same letter are significantly different (*p* < 0.05).

**
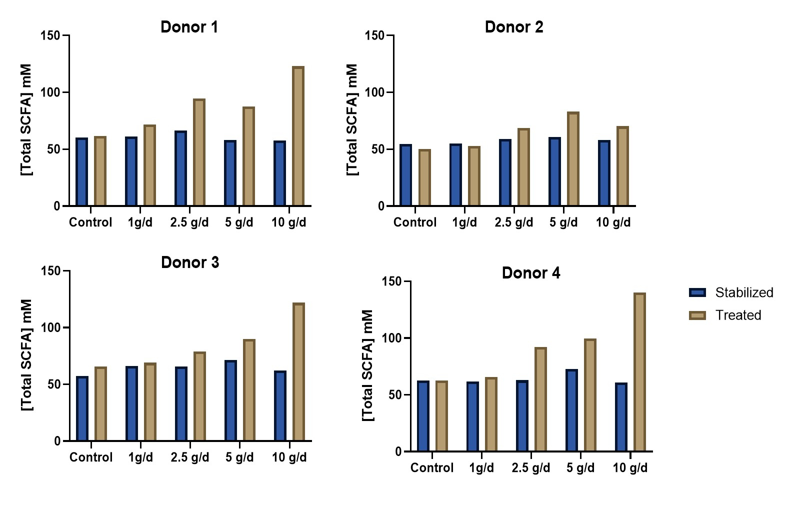
**

**Figure S6:** Individual donor responses in total SCFA concentrations induced by scFOS. Concentrations of total short-chain fatty acids (SCFAs; mmol·L⁻¹), including acetate, propionate, and butyrate assessed at day 7 (stabilized) and final days (average of days 19 to 21 = treated). Values are shown as means per donor and treatment condition.


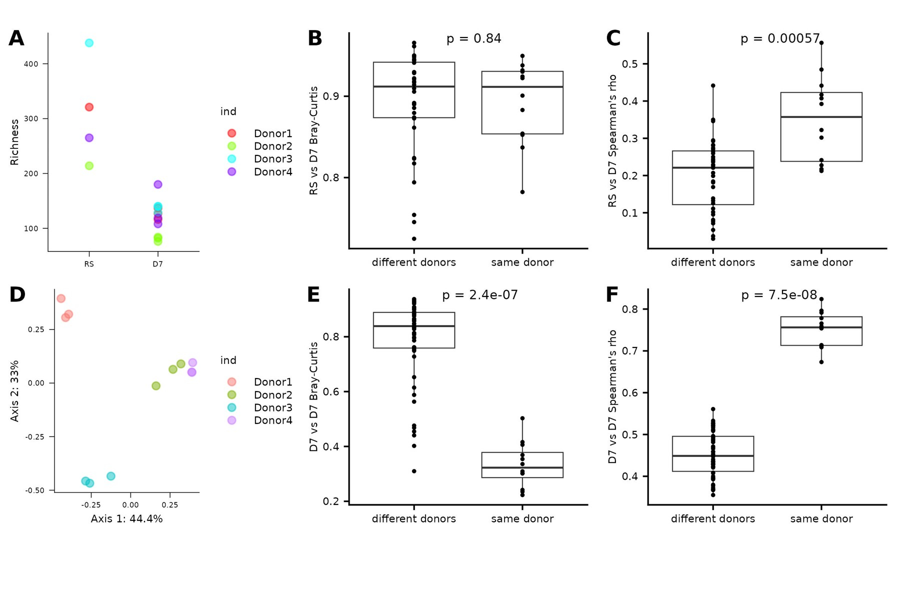


Figure S7: Comparison between RSs and D7 samples. (A) Species richness of RSs and D7 samples, colored according to donor. (B-C) Distribution of (B) Bray-Curtis dissimilarity and (C) Spearman’s correlation computed between RSs and D7 samples, according to sample relationship (same donor or different donors). P-values from Wilcoxon tests are shown. (D) PCoA based on Bray-Curtis dissimilarity computed on D7 samples only. Samples are colored according to donor. (E-F) Distribution of (E) Bray-Curtis dissimilarity and (F) Spearman’s correlation computed between D7 samples, according to sample relationship (same donor or different donors). P-values from Wilcoxon tests are shown.

**
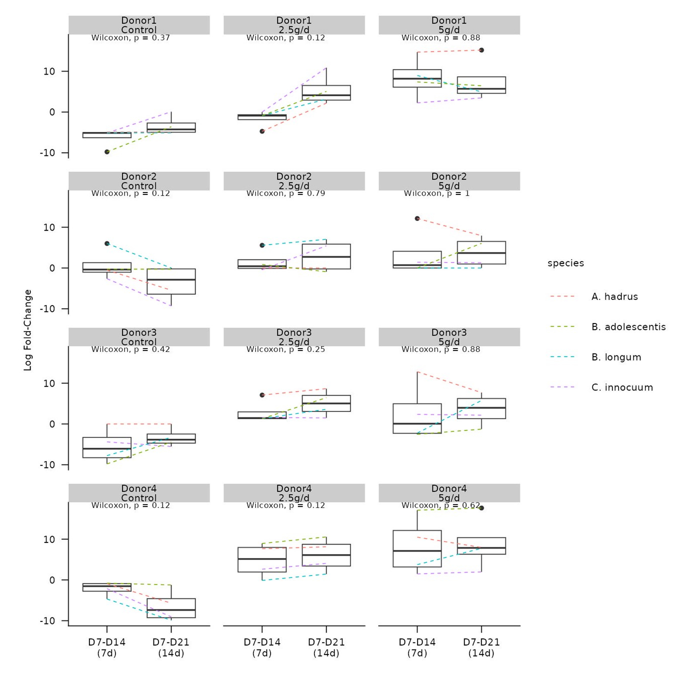
**

**Figure S8**: Log-fold change of increasing species.

Log fold-change between D7 and D14 or D7 and D21 for the 4 species significantly increased by scFOS, in each fermenter and each donor. Dashed lines connect points corresponding to the same species. P-values from Wilcoxon signed-rank tests are displayed. *A. hadrus = Anaerostipes hadrus; B. adolescentis = Bifidobacterium adolescentis; B. longum = Bifidobacterium longum; C. innocuum = Clostridium innocuum.*


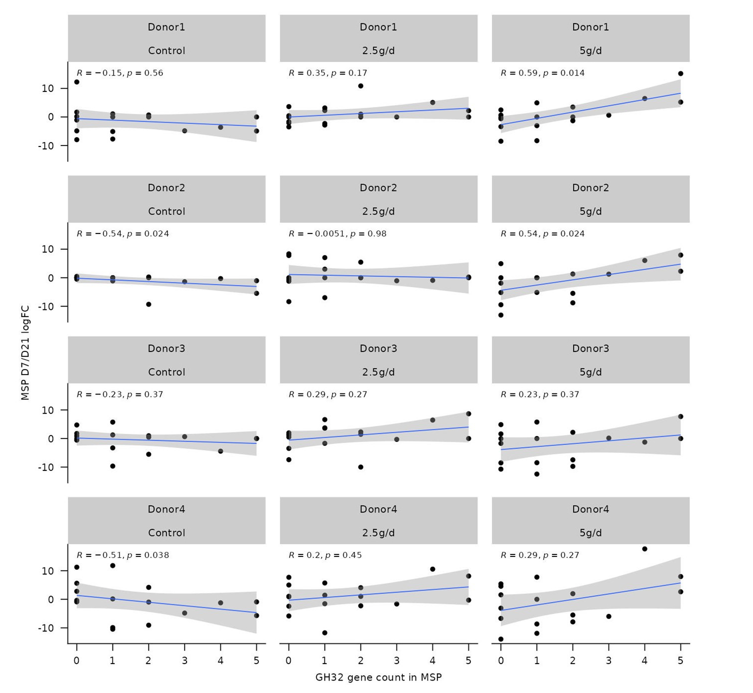


**Figure S9**: Relation between GH32 content and log fold change of impacted species.

Relation between GH32 content and log fold change of impacted species: Donor-specific log fold-change between D7 and D21 of the 17 significantly impacted species, according to the number of genes annotated as GH32 in their pangenome. Spearman’s correlation coefficients along with their associated *p*-value are displayed.


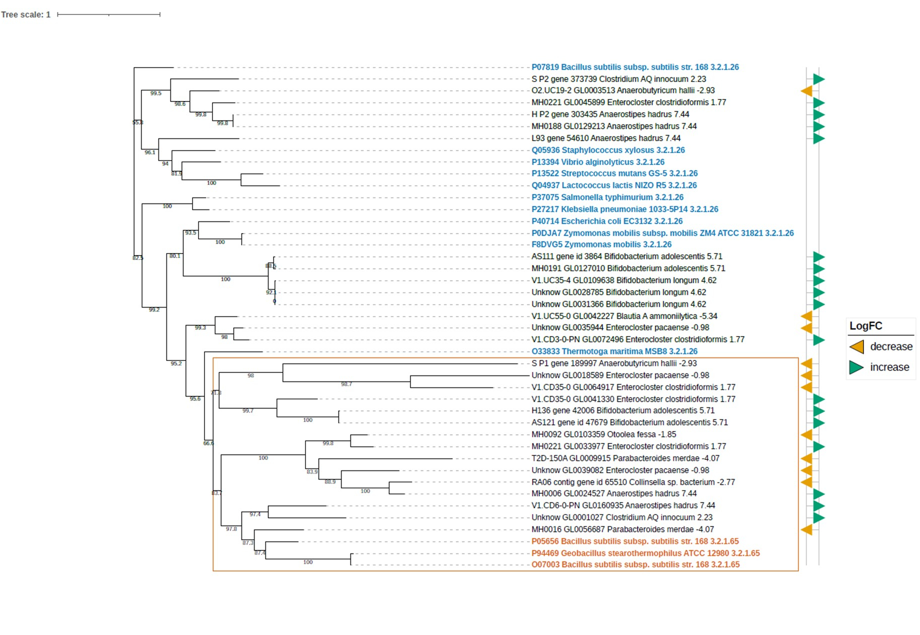


Figure S10: Phylogenetic tree of GH32 genes. Phylogenetic tree of 29 proteins from the gut catalogue annotated as GH32 and belonging to one of the 17 species impacted by scFOS. Known GH32 proteins with β-2,1 linkage activity (labeled in blue) or β-2,6 linkage activity (labeled in red) were included. Triangles on the right indicate whether the corresponding species increased or decreased in the 5 g/d fermenter at D21. The red rectangle highlights the clade of proteins predicted to have β-2,6 linkage activity, while all other proteins are predicted to have β-2,1 linkage activity. Numbers on branches indicate bootstrap support values (percentage).


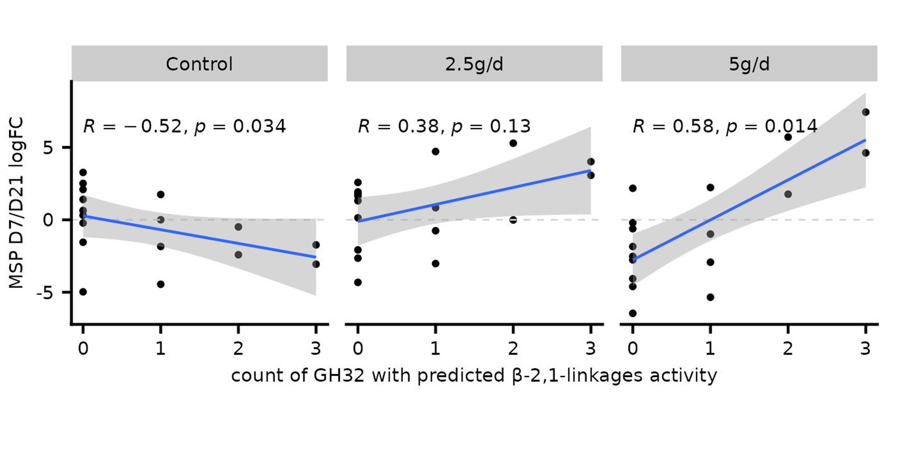


Figure S11: Species **log fold-change and gene content.** Relation between log fold-change between D7 and D21 for the 17 species significantly impacted by scFOS, and the number of genes annotated as GH32 and predicted to have β-2,1 linkage activity in their pangenome. Log fold-change values represent the median across the four donors. Spearman’s correlation coefficients along with their associated p-value are displayed.
